# Urdu Translation and Linguistic Validation of the Bladder Cancer Index Questionnaire

**DOI:** 10.7759/cureus.27487

**Published:** 2022-07-30

**Authors:** Arsalan Pervaiz, M Hammad Ather, Ahmad Bashir, Wajahat Aziz

**Affiliations:** 1 Section of Urology, Department of Surgery, Aga Khan University Hospital, Karachi, PAK; 2 Section of Urology, Department of Surgery, Aga Khan University, Karachi, PAK

**Keywords:** urdu, bladder cancer index questionnaire, translation and validation, quality of life (qol), bladder cancer

## Abstract

Background

This study aimed to translate the Bladder Cancer Index (BCI) questionnaire to Urdu and validate it to assess the quality of life of patients with bladder cancer.

Material and methods

After forward and backward translation of the BCI questionnaire into Urdu, content validity was calculated using the content validity index (CVI) based on input from five health experts regarding the clarity and relevance of the questionnaire. Construct validity was measured by comparing the inter-scale domains and subdomains of BCI and by comparing BCI with Short Form 36 (SF-36) using correlations. For assessment of reliability, Cronbach’s alpha was calculated to measure internal consistency and for test-retest reliability, the questionnaire was re-administered four weeks later and the correlation of responses at baseline and at a four-week time point was evaluated.

Results

The questionnaire has good content validity for clarity (0.91) and relevance (0.87). The construct validity of BCI was also adequately displayed by moderate to high correlation between different subdomains of BCI (Pearson’s r: urinary - 0.62, bowel - 0.78, sexual function - 0.43) and low to moderate correlation between responses of BCI compared with SF-36 (Pearson’s r mostly >0.45). Test-retest reliability was excellent (Pearson’s r 0.90-0.98), and there was good internal consistency (Cronbach’s alpha 0.79-0.92) in the different domains of the questionnaire.

Conclusion

The Urdu-translated BCI is a valid and reliable tool to measure the impact of bladder cancer on the quality of life of patients.

## Introduction

Bladder cancer (BC) is the seventh most commonly diagnosed cancer in the male population, and it is the second most common urological malignancy worldwide [1]. In Pakistan, it is the eighth most common cancer diagnosed among males [1].

A major proportion of bladder cancers (up to 75%) are non-invasive, requiring long-term follow-up with cystoscopy and surveillance [2]. This may significantly affect the quality of life. On the other hand, the muscle-invasive disease requires extensive surgery such as radical cystectomy with the formation of a neobladder, continent catheterizable pouch, or ileal conduit. This may particularly affect physical and social functioning. Bladder cancer, whether invasive on non-invasive, puts a high toll on the patient in terms of both quality of life and finances. BC patients experienced a statistically significant decline in Health-Related Quality of Life (HRQOL) in all domains. For evaluating the impact of a certain disease process as well as monitoring the side effect and benefits of the treatment, HRQOL is an important outcome [3]. Quality of life in bladder cancer patients can be measured by the different quality of life questionnaires [4]. Qualitative measures are routinely used, however, quantitative measures have also been validated in this patient population. A number of HRQOL questionnaires have been developed either by the European Organization for Research and Treatment of Cancer (EORTC) or the American organization for Functional Assessment of Cancer Therapy (FACT). The EORTC-QLQ-BLS24 and FACT-bladder modules measure the quality of life in noninvasive bladder cancer while the EORTC-QLQ-BLM 30 and FACT Vanderbilt cystectomy index are commonly used for muscle-invasive bladder cancer [5-7].

The Bladder Cancer Index (BCI) questionnaire was developed in 2007 by Gilbert et al. to assess the quality of life of patients with bladder cancer (Appendices). It is a comprehensive questionnaire that is neither treatment nor gender-specific and is applicable to patients suffering from either muscle-invasive or noninvasive bladder cancer [8].

This questionnaire has earlier been translated and validated in the Spanish, Arabic, Dutch, Hungarian, Japanese, and French dialects [9-14]. However, to date, no validated Urdu version of this questionnaire is available to assess the quality of life of bladder cancer patients. Although Short Form 36 (SF-36) is similar to BCI, it is not as comprehensive or specific as BCI (Appendices) [9].

## Materials and methods

Development of the Urdu version of the BCI questionnaire

We used the standard translation and back-translation methods. Two urologists with experience in diagnosing and treating men with bladder cancer individually translated the original English version of the BCI questionnaire into Urdu (Appendices). To further refine translation, a pilot study among four patients (three patients with non-muscle-invasive bladder cancer and one with muscle-invasive) was done by carrying out cognitive debriefing interviews to assess understandability and to highlight any discrepancies with the original version. As the translated version was quite understandable, only minor changes were made, and a backward translation from Urdu to English was performed to ensure that during the reverse translation, there was no major discrepancy with the original version.

Validity

Content validity was calculated by recording the responses of five health experts. The finalized Urdu version of the questionnaire was sent to five health experts, and they were requested to assess every item on a 4-point Likert scale (1 = not relevant, 2 = only relevant if the phrasing is strongly adjusted, 3 = relevant with some adjustment to phrasing, 4 = very relevant). The content validity index (CVI) was calculated by dividing the average score given by experts by the maximum score possible.

Construct validity was assessed by comparing different BCI subdomains and by comparing them with SF-36.

Hypothesized results were a moderate to high correlation between function (measuring severity) and bother (impact) of symptom, and a moderate to low correlation between BCI and SF-36. Correlations of <0.45 were considered low, 0.45-0.70 moderate, and >0.70 high [15].

Reliability

To investigate test-retest reliability, the BCI questionnaire was administered twice, once at baseline and once again four weeks later, and the responses were compared. Internal consistency was calculated using Cronbach’s alpha for all the domains and subdomains of the BCI questionnaire.

Data collection

After ethical review committee (ERC) approval, all patients fulfilling inclusion criteria were enrolled from July 2020 till December 2020, and informed consent was taken. Data were collected for demographics, including age, gender, comorbid conditions, marital status, educational level, smoking history, and cancer-related factors.

HRQOL was assessed before and one month after treatment. A total of three questionnaires were filled out by each patient. At the initial presentation, patients were asked to fill both the Urdu version of the BCI questionnaire as well as SF-36 to calculate the correlation between different domains of the BCI and SF-36 questionnaires. The BCI questionnaire was again administered four weeks later to assess the retest reliability.

Statistical analysis

Analysis was done using SPSS software (IBM Corp., Armonk, NY) and Microsoft Excel (Microsoft Corporation, Redmond, WA). CVI was calculated. For content validity, CVI >0.7 was considered satisfactory. Construct validity was assessed using Pearson's correlation coefficient (r). The inter-scale domains and subdomains of BCI were compared amongst themselves and were also compared to the domains of SF-36. Correlations of <0.45 were considered low, 0.45-0.70 moderate, and >0.70 high.

Cronbach’s alpha >0.7 was considered reflective of good internal consistency, test-retest reliability was gauged by comparing responses at baseline and four weeks later using Pearson's r, and p<0.05 was considered significant.

## Results

Between July 2020 and December 2020, 40 patients were enrolled, with a mean age of 56.43±.13.20 years. The mean tumor size was 17.47 ± 1.8mm. The mean number of tumors was 1.7±0.22. The majority (38/40) of the patients were male, with 67.5% having a history of smoking. Ninety percent (90%; 36/40) of patients had a non-muscle invasive (Ta/T1) disease on histopathology following transurethral resection of bladder tumor (TURBT). Four (10%) patients underwent radical cystectomy, all with muscle-invasive bladder cancer on TURBT. No variant pathology was noted. All but four out of 40 patients received a single instillation of a chemotherapeutic agent (Mitomycin C). Intravesical Bacillus Calmette-Guerin (BCG) was administered to 13/40 (42.5%) of patients (Table 1).

**Table 1:** Patients demographics and tumor characteristics BCG: Bacillus Calmette-Guerin; TURBT: transurethral resection of bladder tumor

	TOTAL (n=40%/S.D)
AGE (YEARS)	56.43±13.12
GENDER	
MALE	38 (95%)
FEMALE	2 (5%)
SMOKER	27 (67.5%)
SEXUALLY ACTIVE/ STABLE RELATION	24 (60%)
SIZE OF TUMOR (mm)	17.47±1.8
NUMBER	1.7±0.22
SURGICAL PROCEDURE	
TURBT	36 (90%)
RADIAL CYSTECTOMY	4 (10%)
INTRAVESICAL THERAPY	
NO INSTILLATION	9 (22.5%)
MITOMYCIN	14 (35%
BCG	17 (42.5%)
PATHOLOGICAL TUMOR STAGE	
Ta	13 (32.5%)
T1	23 (57.5%)
T2	4 (10%)
GRADE	
LOW	15 (35%)
HIGH	25 (47.5%)
HISTOPATHOLOGY (TRANSITIONAL CELL CANCER)	40 (100%)

The questionnaire was divided into three domains: Urinary (14 questions), Bowel (10 questions), and Sexual (12 questions). These domains were further subdivided into subdomains based on the severity (function) and impact of the symptoms (bother). Mean scores of subdomains of BCI ranged from 44.83 to 91.98 with an observed range between 5.00 and 99.38. Patients reported the worst scores in the Sexual domain (Table 2).

**Table 2:** Distribution of BCI scores and reliability analysis BCI: Bladder Cancer Index

BCI domains	Number of items (n = 36)	Average BCI score (mean ± s.d)	Observed range	Cronbach’s alpha	Test-retest correlation
URINARY	14	80.34 ± 32.75	5.00 – 98.13	0.87	0.98*
Function	6	70.17 ± 38.40	5.00 – 93.30	0.61	0.90*
Bother	8	87.97 ± 25.27	78.13 – 98.13	0.88	0.98*
BOWEL	10	90.05 ± 21.23	81.25 – 99.38	0.79	0.94*
Function	4	87.16 ± 21.33	81.30 – 93.13	0.40	0.93*
Bother	6	91.98 ± 20.98	81.25 – 99.38	0.69	0.93*
SEXUAL	12	58.65 ± 39.50	35.73 – 95.63	0.92	0.94*
Function	7	44.83 ± 38.67	35.73 – 53.75	0.94	0.95*
Bother	5	78.00 ± 31.86	70.00 – 95.63	0.88	0.91*

Content validity

Based on responses from five experts, the CVI was calculated for relevance and clarity. The CVI for relevance was 0.87, and for clarity, it was 0.91, with the universal agreement of the experts on 16 questions for relevance and 21 questions for clarity.

Construct validity

To test the construct validity, the BCI questionnaire was compared to the SF-36 questionnaire using a multimethod matrix of correlation. When the function was correlated with a bother for individual domains, there was a moderate correlation between the Urinary (0.62) and Bowel (0.78) function and bother. However, a weak correlation was observed in the Sexual domain between function and bother (0.43).

When compared to SF-36, there was predominantly a weak correlation, with most correlation coefficients below 0.6. Exceptions included a correlation between the physical symptoms of SF-36 and the Bowel (0.63) and Sexual function (0.70) on the BCI scale (Table 3).

**Table 3:** Correlations among the BCI subscales and SF-36 summary component scores BCI: Bladder Cancer Index; SF-36: Short Form 36

	BCI at baseline						
		URINARY		BOWEL		SEXUAL	
BCI at baseline		Function	Bother	Function	Bother	Function	Bother
	URINARY						
	Function	1					
	Bother	0.62*	1				
	BOWEL						
	Function	0.58*	0.46*	1			
	Bother	0.48*	0.32*	0.78*	1		
	SEXUAL						
	Function	0.45*	0.42*	0.40	0.17	1	
	Bother	0.20	0.47*	0.24	0.19	0.43*	1
SF36							
	PHYSICAL	0.56*	0.47*	0.63*	0.52*	0.70*	0.43*
	MENTAL	0.31	0.54*	0.11	0.05	0.56*	0.47*

Reliability

There was good internal consistency in all domains as demonstrated by a high Cronbach’s alpha (0.79-0.92) with variations observed at the subdomain level (0.40-0.94) (Table 2).

Test-retest at four weeks demonstrated a very strong correlation ranging between 0.90 and 0.98. On comparing different subdomains, a weak correlation was observed between all the subdomains of BCI at baseline and at four weeks except for bowel bother at baseline and bowel function at four weeks (0.76) and bowel function at baseline and bowel bother at four weeks (0.77) (Table 4).

**Table 4:** Correlations among the BCI subscales at baseline and after four weeks BCG: Bacillus Calmette-Guerin

BCI at 4 weeks	BCI at baseline						
		URINARY		BOWEL		SEXUAL	
		Function	Bother	Function	Bother	Function	Bother
	URINARY						
	Function	0.90*	0.53*	0.56*	0.49*	0.54*	0.23
	Bother	0.58*	0.98*	0.45*	0.31	0.45*	0.46*
	BOWEL						
	Function	0.56*	0.44*	0.91*	0.76*	0.46*	0.17
	Bother	0.48*	0.32*	0.77*	0.93*	0.28	0.15
	SEXUAL						
	Function	0.46*	0.40*	0.43*	0.25	0.95*	0.26
	Bother	0.27	0.51*	0.32*	0.26	0.45*	0.91*

## Discussion

BCI is a comprehensive questionnaire that assesses the severity of symptoms and their impact on quality of life. It has three domains: Urinary, Bowel, and Sexual. Each domain is further subdivided into a functional and bothers subdomain. It was translated into Urdu and validated for its use in bladder cancer patients. Validation was done using content and construct validity, and reliability was evaluated by measuring internal consistency and test-retest reliability.

Similar to the Arab and Dutch studies, content validity was very good, with a reported CVI of 0.87 and 0.91 for relevance and clarity, respectively [13-14]. Construct validity was investigated by comparing the Pearson coefficients of the BCI domains and subdomains to a comparable questionnaire, SF-36. Since bother directly reflects the impact of symptoms on function, the two are expected to have a moderate to high correlation. A high correlation was seen in the Bowel (r=0.73) and moderate in Urinary (r=0.62) domains. However, a low correlation was reported in the Sexual domain (r=0.43). This is in line with a study conducted earlier, except reporting high correlation in the Bowel domain as opposed to moderate correlation reported previously [9]. When the subdomains of BCI were compared to SF-36, mostly moderate to low correlations were reported. Low correlations were mainly reported when comparing the Mental domain of SF-36 to the subdomains of BCI. This differs from the low correlations reported by Schmidt S et al. when comparing both the domains of SF-36 with those of BCI [9].

There was good internal consistency as well as test-retest reliability. The internal consistency in all three domains (Cronbach’s alpha: 0.79-0.92) is in agreement with previously reported literature (Cronbach’s alpha: 0.70-0.97) [9-13]. Test-retest correlation at four weeks between the three domains: Urinary, Bowel, and Sexual - were all >0.90, suggesting a very strong correlation as formerly reported and hypothesized [11-12].

We found that the BCI is a useful tool that can be used to assess symptoms and quality of life in patients with bladder cancer, including those undergoing radical cystectomy. As there is a lack of questionnaires in the Urdu language to be used specifically for this population, we could only compare it with a relatively generic SF-36 questionnaire to assess validity. However, despite these limitations and limited sample size, we can confidently state that this questionnaire can be used for patients with bladder cancer to assess the Urinary, Bowel, and Sexual domains.

## Conclusions

A validated Urdu version of the quality of life measure is much needed for bladder cancer patients in our part of the world, where Urdu is the standard medium of communication. The BCI is a multidimensional measure of bladder cancer-specific health-related quality of life. It can assess the health outcome across a range of treatments available for bladder cancer patients.

## Figures and Tables

**Figure 1:**
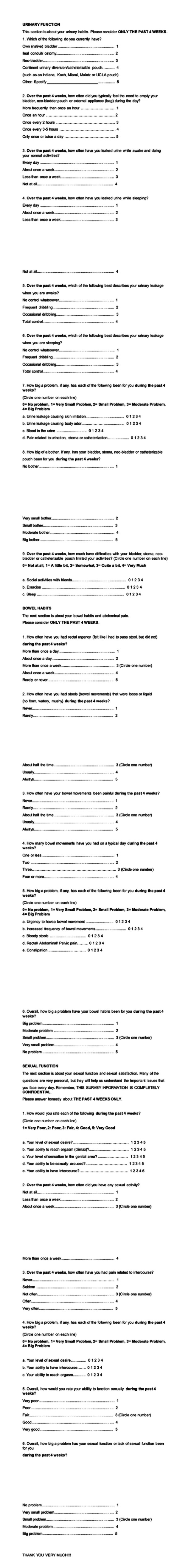
The Bladder Cancer Index (BCI) questionnaire

**Figure 2:**
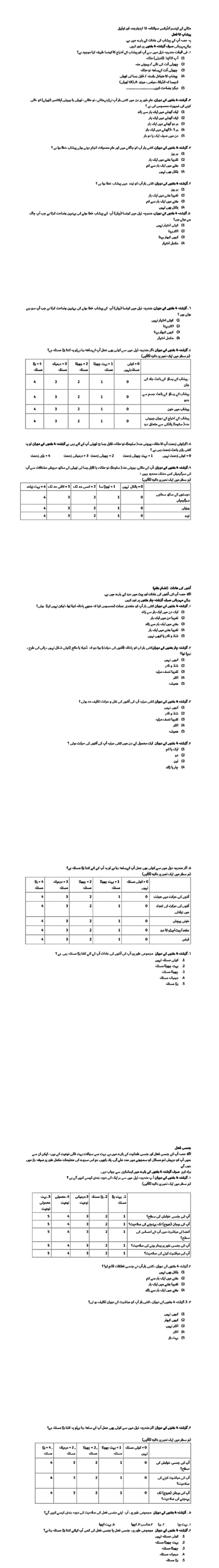
Validated Urdu version of the Bladder Cancer Index questionnaire

**Figure 3:**
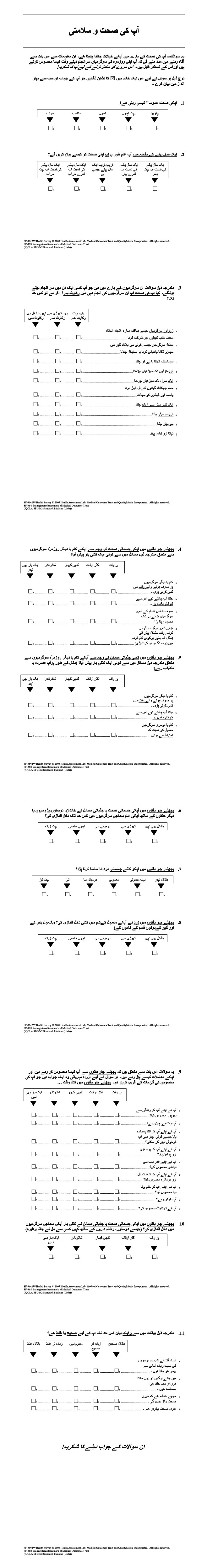
SF-36 Questionnaire
